# Validity and reliability of the perinatal anxiety screening scale in a brazilian sample of pregnant women

**DOI:** 10.1192/j.eurpsy.2021.1617

**Published:** 2021-08-13

**Authors:** M. Barros, M. Aguiar, A. Macedo, A.T. Pereira

**Affiliations:** 1 Departamento De Ciências Naturais, Universidade do Sudoeste da Bahia - UESB, Vitória da Conquista, Bahia, Brazil; 2 Instituto De Psicologia Médica, Universidade de Coimbra, Coimbra, Portugal; 3 Pos Graduação Em Psicologia Da Saúde, Universidade Federal da Bahia, Vitória da Conquista, Brazil; 4 Institute Of Psychological Medicine, Faculty Of Medicine, University of Coimbra, Coimbra, Portugal

**Keywords:** Perinatal Anxiety, validation, pregnancy, Reliability

## Abstract

**Introduction:**

The Perinatal Anxiety Screening Scale was translated and validated for European Portuguese (PASS-29; Pereira et al. 2019), from the original PASS (composed of 31 items; Somerville et al. 2014) to allow epidemiological and correlational research and early detection, which is an health policy imperative. This need also applies to Brazil, where a specific instrument to measure perinatal anxiety is not available.

**Objectives:**

To study the psychometric properties of the PASS Brazilian version factor structure using confirmatory factor analysis (CFA), internal consistency and pattern of correlations with mood states.

**Methods:**

350 women (Mean age: 30.01±5.452) in the second trimester of pregnancy (Mean weeks =25.17±±6.55) completed the PASS and the Brazilian version of Profile of Mood States (POMS-25; Barros et al. 2021). SPSS and AMOS software were used.

**Results:**

After deleting two items (1 and 2) and some errors correlated, CFA indicated a good fit for the second-order model (X^2^/df=2.987; CFI=.903; TLI=.889; GFI=.797, RMSEA=.075; p[rmsea≤0.01]<0.001). The Cronbach alpha was α=.937, and for the four dimensions (general anxiety and specific fear, perfectionism and control, social anxiety and adjustment disorder, acute anxiety and trauma.), were all α>.800. PASS total and dimensional scores significantly (p<.01) and moderately correlated with Profile of Mood States dimensions: negative affect (.471), Depression (.294), Anxiety (.548), Fatigue (.438) and Vigour (-.288).

**Conclusions:**

Similarly, to what has been found for Portuguese version, the Brazilian PASS resulted in a 29-items-and-four-factors version, with good construct and convergent validity and reliability. In the near future we will determine the PASS cut-offs to screen for anxiety disorders in pregnancy and postpartum.

